# On the Phenomena, Causes, and Treatment of Sea-Sickness

**Published:** 1808-05

**Authors:** Edward Miller

**Affiliations:** New York


### *On the Phenomena, Causes, and Treatment of Sea-Sickness.* By Edward Miller, M. D. *of New York.*

(Concluded from our last, p. p. 303.)

SEA-SICKNESS, like many other diseases of association, is greatly under the dominion of emotion, and passions of the mind. By forcibly directing the attention to a particular object, the nausea may be relieved, at least for a short time. By joyful or alarming news, by the terrors of a storm or of shipwreck, by the prospect of battle, and by violent pain, such as the anguish of a broken or dislocated cated bone, the disease may be instantly arrested. But as such degrees of emotion and pain cannot safely be excited on many occasions, and are not susceptible of measure or regulation, they are obviously unfit for practical purposes.

It has been asserted, that keeping the eyes shut or covered, if begun immediately upon embarkation, will prevent sea-sickness. According to the principles maintained in this paper, such an expedient cannot be without use. In a short passage particularly, lying horizontally, sitting or standing, so as to be firmly and steadily supported in one position, with the eyes, for the most part, shut, is by no means impracticable, and deserves to be strongly recommended. I am informed, by a gentleman of observation, that, while at sea, he enjoyed almost total exemption from this complaint during the darkness of the night, and while he lay horizontally, with his eyes closed, but always experienced a return of it the next morning, as soon as he arose, and began to look at surrounding objects. All agree that it is proper to avoid watching or gazing at the waves, especially when they are strongly agitated by tempest.

The proper management of diet will do much, both in the prevention and cure of this disease. It is advised to eat moderately and frequently, to avoid every thing calculated to produce indigestion, and to select such articles as the stomach can digest with the greatest ease, expedition and certainty. For this purpose mariners recommend bread and fresh meat to be eaten cold with pepper; but the occasional use of salted meats will not be found hurtful, and sometimes they undoubtedly deserve a preference. Some contend, that keeping the stomach constantly full, by eating biscuit, &c. is one of the best preventives; and it is not improbable that the stimulus of nutritious and well-adapted food, combined with the stimulus of distention, may be so adjusted as greatly to fortify the powers of the stomach. For drink, it is recommended to use liquids impregnated with the vegetable or carbonic acids; such as lemonade, seltzer-water, sound malt liquors, cyder, champaigne, &c.

The sea-sick are advised to keep much upon deck, even in all varieties of the weather. It is also enjoined upon them to take brisk exercise, such as assisting at the pumps, or any other active employment, with as little intermission as the nature of the case will allow: for it has been generally remarked, that indolent and slothful passengers are most apt to suffer from this complaint. Governor Winthrop, in his Journal, mentions the efficacy of exercise, on a voyage, as a remedy for sea-sickness, in the following terms: "Our children and others that were sick, and lay groaning in the cabbins, were fetched out; and, having stretched a rope from the steerage to the main-mast, we made them stand, some of one side and some of the other, and swing it up and down till they were weary, and by this means they soon grew well and merry.*"

As sea-sickness is undoubtedly a disease of association, and, in that respect, akin to the nature of fevers, it is probable that the stimulant and invigorating remedies employed to repel the attack, as well as to prevent the recurrence of the paroxysms of intermittent fevers, might also be successful in guarding the stomach against the invasion of this complaint. The Peruvian bark and other bitters would be likely to answer this purpose And after the actual attack of the disesse, if great prostration of strength and exhaustion ensue, these remedies, combined with wine and opium, as in fevers of debility, should be assiduously used.

Preserving regularity of the intestinal discharges, and occasionally exciting some degree of artificial diarrhœa, will form an important part of the treatment. The aloetic preparations are among the most suitable of the cathartic class. If acidity be troublesome, as often happens to the feeble and dyspeptic, magnesia will become necessary. Injections of sea-water and soap are always convenient, and deserve to be very frequently employed. It is probable the injection of cold water, or iced water, which, according to Mons. Pomme, so instantaneously relieves the inverted motions of the alimentary canal in hysteria, would likewise prove an efficacious remedy in this case. (See Pomme Des Affections Vaporeuses, p. 25.)

As the stomach and skin are very strongly associated, the former may be often excited into action, and supported, through the medium of the latter. For this purpose the sea-sick may use the warm bath alone, or alternated with cold bath, friction of the skin with oil and camphor, or dry, with powder of mustard: to the epigastric region they may apply plasters, epithems or cataplasms charged with aromatics and opium, and, in severe cases, sinapisms or blisters.

Compression of the abdomen, by means of a bandage or

---

* Winthrop's Journal of the Transactions and Occurrences in the Settlement of Massachusetts, &c.

or handkerchief, is recommended by seamen, and, there is reason to suppose, on good grounds. The savages of North America, when restricted to scanty food, and pressed by hunger, fasten a belt round their bodies; by this they give support to the empty and enfeebled stomach, and thereby provide a substitute for the stimulus and distention of food. When the stomach has been long harassed with the retchings of sea-sickness, this mechanical aid will assist in sustaining the system until it becomes habituated to its new situation.

It will seldom be requisite to combine many of these remedies in the treatment of a single case. For the most part, relief is easily and speedily obtained, and the common method may be delivered in a few words: when nausea comes on, and cannot be subdued by mental exertion, the patient should place himself in a horizontal position, shut his eyes, and lie perfectly still. If vomiting succeed, he should take some draughts of an infusion of chamomile, peppermint or ginger, or something similar. Sea-water is commonly recommended by mariners. When the stomach has been thoroughly emptied by the assistance of such drinks, it becomes necessary to use some grateful stimulant. I am informed, that on board of the packet-boats plying between the British ports and those of the adjacent continent, for the conveyance of passengers, that spiced wine is the common remedy. Where this fails, recourse may be had to small doses of sulphuric (vitriolic) ether, frequently repeated, till it compose the stomach. Small doses of opiates should also be tried. The effervescent mixture of Riverius, seltzer-water, lemonade and warm punch, will succeed in some cases. But if the disease, notwithstanding, should obstinately continue, the stomach be harrassed with incessant retchings, and exhaustion and debility take place to an alarming degree, it will be requisite to adopt the treatment usually pursued in low fevers of debility. Preparations of Peruvian bark, or rather of columbo or quassia, with wine and opiates, or ether, employed in rotation, and periodically repeated, so as to sustain a moderate and equitable excitement of the stomach, will especially claim attention. And, in such extreme degrees of the disease, the other remedies above-mentioned may likewise be selected or combined in such manner as to suit the exigencies of the particular case.

---

It is often necessary to attempt the cure of one disease by exciting another. With this view phthisical patients and and others often are sent to sea. Instead of inquiring into all the circumstances of a sea-voyage, which may prove beneficial in such diseases, it will be sufficient for the present purpose to consider the affection of the stomach as one of the chief means of relief. The instances of the efficacy of this remedy are too numerous and remarkable to admit of a doubt. But it has happened, in many cases, from some peculiarities of the stomach or constitution generally, that the usual nausea and vomiting have not occurred, or have been so slight and transient as to disappoint every hope of advantage from the voyage. If the efficacy of this remedy really depend upon the excitement of nausea and vomiting, it is much to be regretted that such a disappointment should take place; as it seems always to be in the power of the voyager to increase the force and duration of the nausea, by artificial means, to any desirable extent. Swinging, in one form or another, may conveniently be employed in aid of the marine vertigo. If the oscillating or pendulum-like swing should not prove sufficient to create the requisite degree of vertigo, the patient might be whirled in a chair suspended from aloft by two parallel cords hanging near to each other, which, after being circularly revolved fifty or one hundred times in one direction, would return with great velocity in the other. Or, if the debility of the patient should not allow this kind of motion, a small bed, affording room to lie in an easy position, might be suspended to a simple machine, adapted to whirl it with any proper degree of gentleness or velocity. By some of these means, varied in such manner as to suit the circumstances of each particular case, there can be no doubt that vertigo might be increased and regulated at pleasure.

In other cases, likewise, besides phthisis pulmonalis, the marine nausea might be usefully augmented by additional unaccustomed motions. The noted example of its efficacy, mentioned by Mr. John Hunter, in causing the matter of a large bubo to be unexpectedly absorbed, is a proof of great power in promoting the action of the lymphatic vessels. The use of emetics, in chronic diseases, might, perhaps, be entirely superseded by sea-sickness, properly assisted and regulated by some of the other means of exciting vertigo.

